# The Influence of Psycho-social Factors on Participation Levels in Community-based Breast Cancer Prevention Programs in Tehran, Iran

**DOI:** 10.5539/gjhs.v4n1p42

**Published:** 2012-01-01

**Authors:** Maryam Ahmadian, Asnarulkhadi Abu Samah, Ma’rof Redzuan, Zahid Emby

**Affiliations:** Department of Social and Development Sciences, Faculty of Human Ecology, Universiti Putra Malaysia 43400, UPM Serdang, Selangor, Malaysia

**Keywords:** Psycho-social factors, Community participation, Participation levels, Community–based programs

## Abstract

**Background:**

Although significant consideration has been devoted to women participation in breast cancer prevention programs, our understanding about the psychosocial factors which influence participation remains incomplete.

**Method:**

The study applied a quantitative approach based on the cross-sectional survey design and multistage cluster random sampling. A total of 400 women aged 35-69 years, were surveyed at 4 obstetric and gynecologic clinics affiliated to Tehran University of Medical Sciences in Tehran: the participation levels of 86 women who have had a mammogram were analyzed based on their self-efficacy, belief, social influence, and barriers concerning mammography utilization.

**Results:**

Consistent with the study framework, in bivariate analysis, the higher level of women’s participation in breast cancer prevention programs was significantly related to more positive belief about mammography (p< .05), greater social influence on mammography (p< .01) and fewer barriers to mammography (p< .01). Self efficacy (p= .114) was not significantly related to the higher level of participation.

**Conclusion:**

Results suggest that women’s participation levels in breast cancer prevention programs might be associated with the specific psychosocial factors on breast cancer preventive behavior such as mammography screening.

## 1. Introduction

Health promotion encourages individuals and communities to take greater responsibility for their health (WHO, 1978). Women’s involvement in health services or programs such as breast cancer prevention programs is another means of community participation in health. A host of individual and psycho-social factors may constrain community participation and its levels.

Previous literatures have documented the influence of individual and structural factors on community participation process. For example, numbers and types of community participants are influenced by geography (Cohen & Syme, 1985), socioeconomic status (Sills, 1968; Widmer, 1987), gender (Wells, *et al.*, 1990), and group heterogeneity (Litwin, 1986). It was documented that a rise of participation happened across a number of basic socio-demographic (Boyce, 2001). Women in traditional societies have low social status within their families and communities; hence, their ability to make their own decisions is severely limited (Raju and Leonard, 2000). Community members with low level of incomes and educational levels had minimal levels of participation usually as clients and volunteers, and no interest in taking responsibility at project management positions (Boyce, 2001).

In brief, the relations between communities and the different age, gender, ethnicity, and socioeconomic characteristics affect community participation. Different cultures and various socio-demographic factors make community participation faced many obstacles in higher levels of participation.

To date very few studies have been carried out based on theoretical models regarding women’s participation in breast cancer prevention groups (Cameron *et al.*, 2005; Gilbar & Neuman 2002; Guidry *et al.*, 1997). However, most of previous researchers used the health belief model to explain the psycho-social factors that influence the participation in breast cancer prevention groups such as breast cancer support group in the United States (Sherman *et al.*, 2008). Higher levels of women participation in support group were associated with potential benefits of participation in a breast cancer support group, fewer perceived barriers to participation, social influence is caused by friends, family, relatives, oncologist, and medical caregivers, perceived illness severity and consistent support over time (Stvense, 1998; Sherman *et al.*, 2008).

Similarly, at the structural level, social, economic and cultural barriers, and at the individual level, motivation can affect local community participation in health in Uganda (Kapiriri, 2003). Boyce (2001) also found these barriers in the study related to community participation of disadvantaged groups such as poor women, street youth, and disabled persons in health promotion projects in Canada.

Community involvement in the diagnosis and solution of health problems is an old opinion of public health. But listening to the concerns and problems of the community residents or starting where the people stand, are more important to participation levels (Minkler, 1990). Volunteer or community activity in other investigations has been linked to use of support groups and may reflect a broader readiness for social engagement and support (Bauman *et al.*, 1992; Taylor *et al.*, 1986). Of course, volunteer activity is also influenced by functional limitations; individuals with severe illness are less able to participate.

As there are few published reports on influencing factors on women‘s community participation and its levels in health programs, it is not considerably possible to compare findings of this study with other researches. Literatures regarding socio-psychological attributes on community participation levels are relatively limited in theoretical depth and much of this effort is atheoretical. Additional theory-based study would promote the field forward, particularly given the complex selection of factors that might promote or restrain community participation in health programs such as breast cancer prevention programs.

This study provided a closer look at combining three theories such as the Theory of Reasoned Action, Health Belief Model and Social Cognitive Theory to identify psychosocial factors which affect women’s participation in any available community-based breast cancer prevention program or activity in Iran. It intends to identify Iranian women’s problems and implementation of systematic interventions involving individual and communities to improve sustainable breast cancer prevention program development.

This paper attempts to understand psychosocial factors such self efficacy, social influence, belief and barriers which affect women’s participation in a community-based breast cancer prevention program or activity in the subgroup of women who were adherent to mammography in last two years. It is the only feasible way to understand effective factors on the higher level of women’s participation in breast cancer prevention programs. Specifically, we anticipated that greater self efficacy, more positive belief, greater social influence and fewer barriers concerning mammography utilization which could enhance higher level of participation in any available breast cancer prevention program or activity.

### 1.1 Community Participation Levels in Health

To explain women’s participation in community-based breast cancer prevention program or activity, the study argues Rifkin’s view on participation levels in community-based program to assess the actual level of participation in the study population. Rifkin (1991) clarified people can participate in a first level to get the benefits of a health project by receiving health services or education which is most passively. At a second level, local people may participate in program activities. People contribute land, labour or money for a health facility or play an important role as health workers. A third level takes place in implementation, where local people are responsible in a program and decide how to conduct certain activities. A fourth level concerns program monitoring and evaluation. But in all these levels so far, local people are still not involved in program planning or in transferring their own needs and interests into a true grassroots development. Only in fifth or final level, people are instructed to decide about the health programs which should be undertaken and ask health staff, agencies and/or the government to provide the necessary expert knowledge and/or resources (Rifkin, 1991). The five levels of participation are as follows:

(1) Health benefits whereby communities are only health or education services users;
(2) Program activities where local communities contribute labour, land or money;
(3) Implementation that focuses on local people’s managerial responsibilities to carry out the program;
(4) Monitor and evaluation of program activities;
(5) Deciding on selecting of proper programs to be carried out.

A comprehensive behavioral evaluation of Iranian women on community participation in cancer prevention program has not been undertaken, yet. It is noteworthy to state that community-based breast cancer prevention programs in Iran are related to some informal educational programs which have progressed at the district, and province levels in health centres, work places, voluntary organizations, schools, and hospitals to provide awareness and education on the breast cancer prevention and screening methods at the community level. In other words, these are community- focused programs prepared for health promotion, especially breast cancer prevention along with family planning, maternal health, children vaccination and prenatal care, and other community health programs in Iran. More details related to women’s participation in breast cancer prevention programs in Iran have been recently published elsewhere (Ahmadian *et al.*, 2010).

## 2. Method

### 2.1 Population of the Study and Location

The data for this study consisted of 400 women aged 35-69 years and were selected using multistage cluster random sampling procedure from four obstetric and gynecologic clinics affiliated to Tehran University of Medical Sciences in Tehran, Iran.

Women were classified depending on the mammography participation or non-participation in the past two years into a participant group and a non-participant group, respectively. Participation levels and determinants of community participation in breast cancer prevention program or activity were determined among participant group.

### 2.2 Measures

Most of the questions for instrument were gained and modified from previous literatures which had illustrated high reliability. Besides, a number of questions were developed only for the purpose of this research to direct important concepts which were not addressed in previous studies, such as questions related to community participation levels in health programs.

The health belief model component, the theory of reasoned action components, and the social cognitive theory component were measured on a 5 point Likert scale from 1 to 5 (1=“Strongly disagree” 2=“Disagree” 3=“Moderate” 4=“Agree” 5=“Strongly agree”). In this study 5 self efficacy items included the social cognitive theory and it is women’s confidence in the ability to participate in a mammography. The scores ranged from 5 to 25 with higher scores indicating higher or greater confidence in doing mammography. Barriers are related to the obstacles which cease women from participating in mammography such as attitudinal and logistic barriers. The scores ranged from 15 to 75 with lower scores indicating fewer barriers in doing mammography and 15 barrier items included in the health belief model components.

Belief is related to women’s belief about results of mammography use which is opinionated by the women. This belief can evaluate how positive or negative the attribute of mammography is. The scores ranged from 10 to 50 with higher scores indicating higher or greater belief which has a positive meaning regarding mammography use. 10 belief items and 5 social influence items included in the theory of reasoned action components. Social influence is related to influence from referent individuals such as doctor, nurse, family members, and friends’ opinion, media and others in the medical community which approve or disapprove doing mammography in women. The scores ranged from 5 to 25 with higher scores indicating higher influence from referent individuals in doing mammography.

The community participation levels were measured by dichotomous scale that examined women participation levels whether they participated in any program for breast cancer prevention or not (Yes=1, No=0). Twenty items of the community participation levels included in Rifkin’s perspective on community participation in health programs. Based on some interviews with health care professionals about current women’s participation situation in health programs, most of the questions were specified as level 1 (benefits) and level 2 (activity), four items and eight items, respectively, and the late eight items specified to level 3-5 related to women participation at implementing, monitoring and planning levels.

The instrument for the study revised for content validity by an expert panel, which comprised three social scientist with specialty in community development, two specialized doctors in surgery, an oncologist, a radiologist with specialty in breast cancer diagnosis, two family medicine physicians, two epidemiologist, a professor with specialty in public health. Content validity was also evaluated with a review of the literature. Face validity of the research instrument was done by committee members and some experts in this field.

A modified and developed questionnaire was translated by three health care professionals fluent in both English and Persian. Back-translation of the instrument preserved the content validity of the items. The researcher evaluated the linguistic and cultural accuracy of translation by using an expert panel, particularly an expert translator. The two versions (Persian and English) of the questionnaire were reviewed by a group of experts in breast cancer screening and arrangement was achieved over the translation. More information of the psychometric assets of the scale and the instrument have been lately published elsewhere (Ahmadian *et al.*, 2010).

### 2.3 Data analysis

Data analysis was carried out using Statistical Package for Social Science (SPSS 13). Descriptive statistics such as ranges, frequency distribution, percentages, means and standard deviations were calculated to explain data preliminarily. Bivariate analyses were performed via a series of independent t-tests, analysis of variance (ANOVA), and chi-square tests. Preliminary exploratory data analysis was conducted to appraise for missing values, detect outliers and check for normality. The data went through consistency tests and variable frequency analysis and entered into program. Pilot testing evaluated other attributes such as precision (reliability) and accuracy (validity).

Reliability testing was conducted on a convenience sample of 31 women aged 35 or above. The Cronbach Alpha was tested on each dimension of self-efficacy, belief, social influence and barriers, as well as participation levels. Based on the reliability alpha, the instruments have shown the reliability of Cronbach’s alpha values (from0.72 to 0.96) in this pilot study. The research program attempted to balance increased model fit and content validity. Correlations between the items of each construct examined and high correlations were desirable to establish convergent validity.

As mentioned above, scales were also pretested and evaluated during the pilot test. The lack of standardized tests to measure a number of constructs related to community participation and psycho-social factors influencing breast cancer prevention required the development of scales using Principal Components Analysis (PCA) and Common Factor Analysis (FA).

Exploratory Factor Analysis was used to determine the fundamental influences on the set of observed variables about the nature of four variables (psycho-social factors) which were counted by examining the extent of each observed variable association with a factor (Tabachnick & Fidell, 2001). The factors explained the data through a reduced number of concepts that returned the original set of variables and were used for further statistical analyses (Hair *et al.*, 1995). Principal Components Analysis was applied to describe the psychometric evaluation of instrument for measuring self-efficacy, beliefs, social influence and barriers. Principle axis factoring analysis generated four factors which relate 72% of the variance to the psychosocial items. Barlett Sphericity test was statistically significant, x^2^(595) =19502.704, p = .000, and the variables were highly correlated to one another. Thus, these data were appropriate to conduct factor analysis. Kaiser-Meyer-Olkin sampling adequacy measure was 0.949. Statistical significance was determined at the level 0.05.

The assessment of frequency distribution for each variable, confirmed that the data set had no problems with skewness and kurtosis. Internal consistency reliability analysis was also performed. Finally, a factor analysis using principle axis factoring with varimax rotation was set to further the evaluation regarding psychosocial aspects of the research framework.

## 3. Results

### 3.1 Women’s Community Participation Levels in Breast Cancer Prevention Program

A total of 400 women aged 35-69 years, were randomly chosen using random cluster sampling: 86(21.5%) were assessed as the participant group and 314(78.5%) as the non-participant group. Table 1 shows the levels of participation in community-based breast cancer prevention programs or activities which were achieved by the participant group (n=86). Table 1 also shows that the women’s participation in breast cancer prevention programs were divided into two levels (benefit, activities). By means of χ^2^ test there is a significant difference in frequency between women who were in only level 1(benefits) and those in level 2(activities) [χ^2^ (1) 26.79, p< .01]. Higher levels (implementing, monitoring, evaluation and planning) were yet controlled by health care professionals in Iran

**Table 1:** Levels of Women Participation in Community-Based Programs (n=86)

Levels	N	(%)		χ^2^	df	P
Level 2	67	77.9		26.79	1	0.01*
Level 1	19	22.1				

Note:

* p<0.01.

### 3.2 Demographic Differences between Women’s Level 1 and Level 2

In this study, selected demographic variables such as age, education, occupation, marital status, and income between the level 1 (benefits) and level 2 (activities) were compared using chi-square (Table 2). The study results showed that the largest proportion of participants in level 2 (activities) were those in the 41 to 45 year age bracket (43.3%), where as the largest rate of the women in level 1(benefits) were older than 51 years old (31.6%). Generally, women in level 2 (activities) tended to be younger. The difference in age between the two levels was significant (χ^2^=14.65, p= .002).

**Table 2:** Demographic Characteristics of participant Group (Levels of women’s Participation in any Community-Based Breast Cancer Prevention Program)

		Only level1 n=19(22.1%)		level2 n=67(77.9)		X^2^	sig
		%	n	%	n		
**Age**	<40	21.1%	4	23.9%	16	14.65	.002*
	41-45	31.6%	6	43.3%	29		
	46-50	15.7%	3	29.9%	20		
	>51	31.6%	6	3.0%	2		
**Education**	Primary &secondary school	21.1%	4	-	-	30.82	.001*
	diploma	36.8%	7	6.0%	4		
	Graduate	42.1%	8	79.1%	53		
	postgraduate	-	-	14.9%	10		
**Marital**	Married	78.9%	15	65.7%	44	1.459	.482
	Widow/divorced	10.5%	2	11.9%	8		
	Single	10.6%	2	22.4%	15		
**Occupation**	Full time Employee	36.8%	7	76.1%	51	23.66	.001*
	Part Time Employee	10.5%	2	17.9%	12		
	Unemployed or Housewife	52.7%	10	6.0%	4		
**Income**	low	10.5%	2	1.5%	1	3.648	.161
	middle	73.7%	14	83.6%	56		
	high	15.8%	3	14.9%	10		
**Insurance**	public	100.0%	19	86.6%	58	2.851	.091
	private	-	-	13.4%	9		

Note:

* p<0.001.

In general, a greater percentage of participating women at both levels were graduates. The table 2 indicates 79.1% for level 2 (activities) and 42% for level 1 (benefits) are women graduates. Furthermore, the difference in education was significant between the two levels (χ^2^=30.82, p=.001). The study showed that greater percentage of women in both levels are married and the difference in marital status was not statistically significant (χ^2^=1.45, p= .482).

The largest proportion of the women in the level 2 (activities) were full-time employees (76%), whereas the largest proportion of women in the level 1(benefits) were unemployed or housewives (52.6%). The statistics shows that participating women in both levels were significantly different in occupation (χ^2^=23.66, p= .001). Table 2 presents a greater percentage of participating women in level 2 (activities) related to the middle class (83.6%). It also indicates that more participants in level 1(benefits), were in the middle class (73.7 %). The difference in income was not significant between 2 levels (χ^2^=3.64, p= .161). Furthermore, insurance status has not showed a significant difference between the two levels (χ^2^=2.85, p= .091).

Notably, however, all participant (n=86) have participated in some programs related to breast cancer prevention, suggesting that most women acknowledged that they seldom participated as an audience in the selected programs. To get better understanding of community participation in breast cancer prevention program among Iranian women, one explanation is that the community participation is referred to some of their activities which embedded into a comprehensive health program on women’s health like family planning at the districts level.

### 3.3 Psychosocial Factors and Its Comparison with the Levels of Community Participation

Independent-sample t-test was conducted to distinguish the influencing factors between the two levels of participation regarding community-based breast cancer prevention program or activity. It was conducted to provide more information regarding self-efficacy among participant women. There was not a significant diversity in self-efficacy between level1 and level 2[t (19.84) =2.39, (p=.114)]. However, the mean self- efficacy score was higher for level 2(M =23.01, SD=2.09) comparing level 1(M=20.21, SD=4.98). It reveals that participant women who were in level 1 (benefit) and level 2 (activity) had no significant difference in self-efficacy in relation to mammography (Table 3).

**Table 3:** Comparing Self efficacy Scores between Two Levels by Using Independent -Sample t-test (n=86)

Self Efficacy	N	Mean	SD	t	df	P
Level 2	67	23.01	2.09	2.39	19.84	.114
Level 1	19	20.21	4.98			

Note: * p <0.05.

#### 3.3.1 Belief and Levels of Community Participation 

Independent sample t-test also was employed to provide more information regarding beliefs among the participant women on the subject of participation levels in programs (Table 4). There was a significant difference in beliefs regarding mammography utilization between women in level1 and level2; [t (84) =2.590, p= .011]. The mean beliefs score was higher for level 2(M =40.38, SD=3.02) comparing level 1(M=38.36, SD=2.90). It states that the women who have participated in the level 2 (activity) had greater belief in doing mammography than those who participated in the level 1 (benefit).

**Table 4:** Comparing Beliefs Scores between Two Levels by Using Independent -Sample t-test (n=86)

Beliefs	N	Mean	SD	t	df	P
Level 2	67	40.38	3.02	2.590	84	0.011*
Level 1	19	38.36	2.90			

Note:

* p<0.01.

#### 3.3.2 Social Influence and Levels of Community Participation

Table 5 shows that the mean score for social influence in level 2 was higher than level 1 with a significant variation; [t (20.371) =3.814, p= .001]. The mean social influence score was higher for level 2(M =20.22, SD=1.36) comparing level 1(M=17.63, SD=2.87). It demonstrates that the women who have participated in the level 2 (activity) had greater social influence regarding mammography utilization than those who participated in the level 1(benefits).

**Table 5:** Comparing Social Influence Scores between Two Levels by Using Independent -Sample t-test (n=86)

Social Influence	N	Mean	SD	t	df	P
Level 2	67	20.22	1.36	3.814	20.371	.001
Level 1	19	17.63	2.87			

Note: * p<0.001.

#### 3.3.3 Barriers and Levels of Community Participation

Table 6 illustrates that higher mean for the barrier in level 1 (mean =40.52, SD=7.49) than the mean barrier in level 2(mean =33.95, SD=2.85); [t (19.50) =-3.74, p= .001]. It means that women should overcome their barriers to mammography for higher level of participation in any community-based breast cancer prevention program. It presents that the women who have participated in the level 2 (activity) had lower barriers in doing mammography than those who have participated in the level 1 (benefit).

**Table 6:** Comparing Barrier Scores between Two Levels by Using Independent -Sample t-test (n=86)

Barrier	N	Mean	SD	t	df	P
Level 2	67	33.95	2.85	-3.74	19.50	0.001*
Level 1	19	40.52	7.49			

Note:

* p<0.001.

## 4. Discussion

This study was conducted on 86 Iranian women (participant group) who have had a mammogram within the last two years, attending at 4 obstetric and gynecologic clinics in Tehran to identify significant psycho-social factors influencing their participation levels in breast cancer prevention programs.

The levels of participation which were achieved by the participant group (n=86) were divided into two levels; level 1 (benefit) and level 2 (activities). Based on the findings of this study it can be concluded that, the levels of women’s participation are limited.

### 4.1 Demographic Factors and Women Participation Levels

There is little published information regarding the relationship between socio-demographic characteristics and community participation levels in health programs, particularly in breast cancer prevention. In this study, the chi-square (χ^2^) test exposed a significant relationship between age, education, occupation and higher level (activities) of participation (P < .01). Furthermore, the study showed marital status (p= .482), insurance status (p= .91) and income (p= .161) have no statistical difference between level1 (benefits) and level 2(activities) among women who participated in any community–based breast cancer prevention program. With respect to demographic variables, one study discovered that socio-demographic characteristics of mothers are associated with the choice to participate in community activities regarding their children health in Indonesia (Nobles, 2006). Another study also demonstrated that low rates of participation of disadvantaged persons in health programs have been characteristic of low socioeconomic status resulting in poor motivational levels Boyce (2001). It is noteworthy to state that numbers and types of community participation are influenced by geography (Cohen & Syme, 1985), socioeconomic status (Sills, 1968; Widmer, 1987), and gender (Wells *et al.*, 1990).

The findings from this study showed that older women have participated abundantly in breast cancer prevention program in level one (benefits) than level 2 (activity). Contrary to the result, association between age and participation in cancer support group was not significant in previous study (Sherman, 2008). However, in general, younger women tend to participate in community-based cancer prevention or family planning program in Iran. This again brings to light the better education and employment of these women in this study.

The findings showed that educated women have participated in breast cancer prevention program or activity in higher level (activity) in comparison to non-educated ones. The number of women with high academic qualification has considerably increased in Iran, so it can be a good evident to support the result. Consistent with the result, relationship between education and participation in cancer support group was significant in previous study and participants had marginally higher education than nonparticipants (Sherman, 2008). Similarly, ‘established’ participants were mostly educated with regard to the participation in a breast cancer support group in the United of States (Stvense, 1998). Several prior studies noted that there were trends for greater participation in cancer support group among those with greater education (Bauman, *et al.*, 1992; Eakin & Strycker, 2001; Meyer & Mark, 1995; Stevens & Duttlinger, 1998).

The result of this study also showed that occupation enables women to decide their own breast cancer prevention program in Iran. It can be concluded that women with full-time jobs have less socioeconomic dependency. Thus, occupation encourages their participation in health promotion programs such as breast cancer prevention programs. Having a job can improve women participation at a higher level (activity). These women can be informed more easily of the relevant medical programs such as those many available work-place programs in Iran. Besides, they are less conservative than the housewives and unemployed women. The employed women can conceive this reality that their own health is equal to the whole family health. In contrast, association between occupation and participation in cancer support group was not significant in previous study (Sherman, 2008).

It is believed that socio-demographic factors are important to community participation levels in breast cancer prevention program or activity but it might not be robust predictors. The time of doing mammography and women’s involvement in the treatment and diagnosis process also affect women’s participation in the breast cancer activities. In this study, the subsample of individuals who have participated in breast cancer prevention program or activity was modest, which may restrict conclusions.

### 4.2. Psychosocial Factors and Women Participation Levels

Results showed that the higher level of participation (level 2) was significantly related more positive belief (p< .05), greater social influence (p< .01) and fewer barriers (p< .01) towards mammography. Self efficacy (p= .114) was not significantly associated to the higher level of participation (level 2).

The study revealed that self-efficacy is not a salient factor for the levels of participation among participant group. In contrast, another study indicated that individuals with low self-efficacy regarding their health behavior restrict their participation in rural NGOs in India (Handy & Kassam, 2004).

It seems self-efficacy did not influence women’s participation in community-based breast cancer prevention programs due to the lower levels of their participation in the limited expected benefits and some activities in Iran. It means that personal factors such as self efficacy cannot influence individual’s willingness to participate in community activities, particularly on health matters. Based on our personal observation, Iranian people are more successful in individual activities than working in a community even in the case of health matters. On another note, participating women have not experienced a strong sense of community in the study, since there is no actual formal program regarding breast cancer prevention in Iran.

Self-efficacy of participating women in breast cancer prevention programs may be slowed down because of women’s limited positions in those programs in Iran. Thus, it causes difficulty in behavior changes such as voluntary participation in breast cancer prevention program. Health care professionals should have knowledge on behaviors modification in order to enhance women’s self efficacy towards voluntary participation in formal or informal breast cancer prevention program.

Results of bivariate analysis showed women who have participated in the level 2 (activity) had greater belief in doing mammography than those who participated in the level 1 (benefit). Consistent with this finding, previous studies showed that positive belief causes people to control the disease individually and to increase their voluntary participation in malaria control activities in Iran (Zaim, 1997; Grantham 2009).

Iranian women specially the traditional and non-educated ones resist against new health behavior and to some extent they refuse to listen to the health care professional’s advice. Similarly, previous study showed that specific cultural belief had association with voluntary participation in health program (Boyd-Franklin, 1991; Guidry, *et al.*, 1997; Mathews, Lannin, & Mitchell, 1994).

The results of this study demonstrated that participant women in the level 2 (activity) had greater social influence regarding mammography utilization than those who participated in the level 1(benefits). Consistent with this result, higher levels of women’s participation in support group were associated with social influence (Stvense, 1998). Similarly, social influence is important to facilitate community involvement at the community level in community-based HIV prevention in Uganda (Leonard *et al.*, 2001). The findings from this study showed that social influence mobilized women to reach higher level of participation in any formal or informal breast cancer prevention programs among Iranian women.

The finding also indicated that women who have participated in the level 2 (activity) had lower barriers to mammography than those who have participated in the level 1 (benefit). Consistent with the results of this study concerning individual barriers and participation in community-based program, previous research showed that some individual barriers such as lack of motivation, time, language, economics, social support, family or household responsibilities, socio-cultural (fear) and environmental (traffic-related) barriers which reduced people attendance in community-based programs (Martinez *et al.*, 2001). Likewise, participation in cancer support groups was associated with practical barriers, access issues and lack of social support and these items are lower in active participant (Sherman, 2008).

To the best of our knowledge, this study is the first quantitative study that shows how the levels of women’s participation in breast cancer prevention program or activity were influenced by psycho-social factors. Earlier researches on community participation levels were mainly qualitative, as a result of which the extent to which participant were influenced by the psycho-social factors could not be determined.

Community-based prevention program is an approach to health promotion and disease prevention that needs high level of motivation for the people to participate. Such motivation comes from a preventive health seeking behaviour of the target population. Women would be active and participate in higher levels of participation in breast cancer prevention programs in an encouraging condition.

As shown in this study, people’s socio-psychological aspects influence participation behaviour. As such, using socio-psychological theories can adjust behavioural aspects of health among individuals, and communities. It had been quite well documented that psychosocial factors made individual and community involve more actively in health programs such as HIV programs, chronic disease, child and maternity and family planning. This proves the importance of a sustained breast cancer prevention program using theoretical-based interventions.

It is obvious that engaging the community in different activities in health programs have many benefits but different cultures, and psycho-social characteristics influence different levels of community participation. Carrying out this study, we have demonstrated that more positive belief, greater social influence, and lower barriers toward preventive health behavior (e.g. mammography use) can motivate women to be involved in health activities.

### 4.3 Limitations of the Study

The current study is a cross sectional survey developed within the positivist approach to social science. Thus, the conclusions are only descriptive in characteristics and do not confirm causal relationships between the variables. Researcher needs to do multiple methods to strengthen the study design, but survey is a basic approach in this study. In addition, Iranian people are very interactive. Thus, more qualitative research with focusing on in-depth interview and focus groups with respondents of study is needed to support this study. Furthermore, the lack of participation of potential key informants is obvious in present research.

Literatures regarding socio-psychological attributes on community participation levels are relatively limited in theoretical depth and much of this effort is atheoretical. The present study cannot be addressed directly to previous literature review. However, the factors influencing active participation in health program can be concluded by highlighting the importance of community participation in health in literature review. Besides, the subsample of individuals who have participated in mammography was reasonable in present study which may limit the conclusions.

These data may be overestimated due to social desirability response bias. Moreover, the present study intends to carry out on a small sample of women and thus our findings may not be generalized to all Iranian women. Specific researches are needed to study medical problems related to women’s adherence to mammography and its regularity in the broader construct of medical study and its impact on community participation levels in breast cancer prevention programs and activities.

Previous literature showed that traditionally community participation has been assessed in quantitative forms, for example, by asking how many people have come to a meeting or how many people have joined in a community activity. The problem is that presence does not mean participation. People may be present, but have no commitment or understanding of what is going on (Rifkin, 2001). Therefore, it is another limitation for the studies related to community participation purpose and factors affect its levels.

This research tries to study women who are involved in any community-based breast cancer program. However, some of those organizations or programs are not specific to breast cancer prevention and women are involved in various programs which associated to their health issues like family planning. Another challenging issue is the difference between participation and membership. Women might participate as a member in those programs.

In addition, it must be added that there is a general limitation that affects the interpretation of literatures presented here. The studies identified and included were based on a review of the published literature. Studies with negative findings might be less likely to be published and thus less likely to be included in this review. This would result in an overstatement of the effectiveness of the study incorporated selected theories as it is hard to tackle them all in any single study. Despite the limitations, this study is the first attempt to study psycho-social factors influencing community participation levels in breast cancer prevention program or activities among Iranian women.

## 5. Conclusion

According to the findings, this study discovered that social influence, belief and barriers to mammography affect women participation levels in breast cancer prevention programs in Iran. In this study, women who had more positive belief, greater social influence and fewer barriers with regard to mammography were more likely to attend in the breast cancer prevention programs which are available at the district level. Thus, attention to social norms and beliefs towards breast cancer prevention in future interventions can increase women’s voluntary participation in those programs. Contrary to earlier expectations, self efficacy was not significantly related to the higher level of participation.

Additional studies on the relationship between the psycho-social factors on breast cancer and its prevention, and women’s participation in public health programs are needed to improve community participation, and effective health promotion programs. This research could be used as a guide for community participation in participatory research which is now an integral part of Iranian health. The findings of this study also contribute to the existing literature on individual’s health-related behavior theories on understanding community participation in future health programs.

Health care depends on the joining of individual and community health care at the local community level (Van Weel *et al.*, 2008). In this case, psycho-social characteristics of individuals on preventive health behavior may encourage them to participate in community-based health programs which it can increase their decision-making ability about medical issues such as breast cancer.

## Appendixes

**Table:**

Following are some questions that are related to your knowledge about mammography. If you do not know, please choose “I do not know”.				
		Yes	No	I do not know
**1**	**Breast cancer can be cured if it is detected early by screening methods such as mammography.**	**( )**	**( )**	**( )**
**2**	**Women aged 40 and older should have annual mammogram or every two years.**	**( )**	**( )**	**( )**
**3**	**Mammogram can find lumps that cannot necessarily be felt by doctor or by yourself when doing breast self exam.**	**( )**	**( )**	**( )**
**4**	**Although no symptoms exist, mammogram is necessary.**	**( )**	**( )**	**( )**
**5**	**Women younger than 40 years should have a mammogram if they have family history of breast cancer.**	**( )**	**( )**	**( )**

**Table:**

Following are some questions that are related to your attitude towards mammography. It includes factors such as self efficacy, belief and social influence in connection with doing mammography. You must choose only one answer for each question. 1. Strongly disagree 2. Disagree 3. Moderate 4. Agree 5. Strongly agree						
		Strongly Disagree	Disagree	Moderate	Agree	Strongly Agree
**1**	**I am confident I will participate in regular mammograms.**	**( )**	**( )**	**( )**	**( )**	**( )**
**2**	**I am confident I will participate in regular mammograms irresponsible of painful procedure.**	**( )**	**( )**	**( )**	**( )**	**( )**
**3**	**I am confident I will participate in regular mammograms without recommendation from a doctor.**	**( )**	**( )**	**( )**	**( )**	**( )**
**4**	**I am confident I will participate in regular mammograms irrespective of time constraints.**	**( )**	**( )**	**( )**	**( )**	**( )**
**5**	**I am confident I will participate in regular mammograms even if it is expensive.**	**( )**	**( )**	**( )**	**( )**	**( )**

**Table:**

These questions are related to your beliefs about doing mammography. You must choose only one answer for each question. 1. Strongly disagree 2. Disagree 3. Moderate 4. Agree 5. Strongly agree						
If I have mammography in the future it would:		Strongly Disagree	Disagree	Moderate	Agree	Strongly Agree
**1**	**Aware me whether I have cancer.**	**( )**	**( )**	**( )**	**( )**	**( )**
**2**	**Allow me to live longer.**	**( )**	**( )**	**( )**	**( )**	**( )**
**3**	**Be important to my family.**	**( )**	**( )**	**( )**	**( )**	**( )**
**4**	**Mean making time for my health is important.**	**( )**	**( )**	**( )**	**( )**	**( )**
**5**	**Mean having a mammogram is a part of the good overall health care.**	**( )**	**( )**	**( )**	**( )**	**( )**
**6**	**Expose me too many of X-rays.**	**( )**	**( )**	**( )**	**( )**	**( )**
**7**	**Cause pain.**	**( )**	**( )**	**( )**	**( )**	**( )**
**8**	**Help me feel in charge of my health.**	**( )**	**( )**	**( )**	**( )**	**( )**
**9**	**Give me a sense of control over my health.**	**( )**	**( )**	**( )**	**( )**	**( )**
**10**	**Feel uncomfortable.**	**( )**	**( )**	**( )**	**( )**	**( )**

**Table:**

These questions are related to social influence regarding doing mammography. You must choose only one answer for each question. 1. Strongly disagree 2. Disagree 3. Moderate 4. Agree 5. Strongly agree						
		Strongly Disagree	Disagree	Moderate	Agree	Strongly Agree
**1**	**I would seek advice from my doctor or health staff about mammography rather making decision by my own.**	**( )**	**( )**	**( )**	**( )**	**( )**
**2**	**I would follow my family’s advice about my mammography even if I prefer doing something different.**	**( )**	**( )**	**( )**	**( )**	**( )**
**3**	**I would follow my friend’s advice about my mammography even if I prefer doing something different.**	**( )**	**( )**	**( )**	**( )**	**( )**
**4**	**I would follow mass media and people in the news about doing mammography.**	**( )**	**( )**	**( )**	**( )**	**( )**
**5**	**I would follow awareness program from public health center NGOs or work place health promotion program about doing mammography.**	**( )**	**( )**	**( )**	**( )**	**( )**

**Table:**

Following are some questions related to the barriers which influence your participation in mammography. You must choose only one answer for each question. 1. Strongly disagree 2. Disagree 3. Moderate 4. Agree 5. Strongly agree.						
You DO NOT participate in mammography for many reasons.		Strongly Disagree	Disagree	Moderate	Agree	Strongly Agree
**1**	**Cost of mammogram is too much.**	**( )**	**( )**	**( )**	**( )**	**( )**
**2**	**Too hard to figure out where to go for mammogram.**	**( )**	**( )**	**( )**	**( )**	**( )**
**3**	**Lack of transportation to get to a mammography center.**	**( )**	**( )**	**( )**	**( )**	**( )**
**4**	**No one to stay with children or grand children**	**( )**	**( )**	**( )**	**( )**	**( )**
**5**	**Worry the breast X-ray might find cancer.**	**( )**	**( )**	**( )**	**( )**	**( )**
**6**	**Doctor /health provider has not advised to do it.**	**( )**	**( )**	**( )**	**( )**	**( )**
**7**	**Do not think mammography can save our life**	**( )**	**( )**	**( )**	**( )**	**( )**
**8**	**People who perform mammography do not treat patients with respect.**	**( )**	**( )**	**( )**	**( )**	**( )**
**9**	**Too many other things are going on in our lives.**	**( )**	**( )**	**( )**	**( )**	**( )**
**10**	**Worry that mammography might give us cancer.**	**( )**	**( )**	**( )**	**( )**	**( )**
**11**	**Do not think we need mammography.**	**( )**	**( )**	**( )**	**( )**	**( )**
**12**	**No one we know talks about getting breast cancer.**	**( )**	**( )**	**( )**	**( )**	**( )**
**13**	**Media and promotional resources about mammograms do not exist in our neighborhood.**	**( )**	**( )**	**( )**	**( )**	**( )**
**14**	**Breast X-ray cannot change our destiny.**	**( )**	**( )**	**( )**	**( )**	**( )**
**15**	**It makes me embarrassed.**	**( )**	**( )**	**( )**	**( )**	**( )**
**Following are some questions that are related to your participation in mammography, please read carefully and answer them. If you have participated in mammography in last two years, answer the questions.**						
					Yes	No
**1**	**Have you ever participated in mammography in last two years? If yes, when.**				**( )**	**( )**
					**Regular**	**Occasional basis**
**2**	**Do you participate in mammography on a regular or occasional basis?**				**( )**	**( )**
**3**	**What was the main reason for your participation in mammography? Just tick one appropriate reason.**			
**( )**	**a**	**My own decision making about participation.**						
**( )**	**b**	**Symptoms or breast problems.**						
**( )**	**c**	**Recommended to have yearly mammogram.**						
**( )**	**d**	**Doctor ordered mammogram.**						
**( )**	**e**	**Health care sector ordered mammogram.**						
**( )**	**f**	**Media advice.**						
**( )**	**g**	**Friend or acquaintance developed cancer.**						
**( )**	**h**	**Recommendation received from public health centers or, work place health promotion programs, or non-governmental organization regarding breast cancer prevention**						

**Table:**

Following are some questions that are related to your participation levels in any available breast cancer prevention program in any where such as public health center, work place health promotion programs, NGOs, cancer association. You must choose only one answer for each question. 1.Yes 2.No			
		Yes	No
**Level 1**			
**1**	**I have participated as an audience in some of the community-based awareness programs about breast cancer prevention held in one of the places such as health center, work place or NGOs.**	**( )**	**( )**
**2**	**I have followed health care professional’s information which was mentioned in community -based awareness programs towards breast cancer prevention.**	**( )**	**( )**
**3**	**I have consulted with my doctor / health staff regarding breast cancer prevention.**	**( )**	**( )**
**4**	**I have been informed about breast cancer screening methods by health care staff.**	**( )**	**( )**
**Level 2**			
**5**	**I have participated as a member in a breast cancer prevention program.**	**( )**	**( )**
**6**	**I have participated as a speaker in some of the programs about breast cancer prevention which were held in one of the places such as health center, work place or NGOs.**	**( )**	**( )**
**7**	**I have participated as a volunteer in some breast cancer prevention programs.**	**( )**	**( )**
**8**	**I have given consultation, comment or information to others about breast cancer prevention.**	**( )**	**( )**
**9**	**I have met other members outside of program to cooperate with them about breast cancer issue.**	**( )**	**( )**
**10**	**I have contacted other members of my current group in community meetings about breast cancer prevention.**	**( )**	**( )**
**11**	**I have advocated community-based program in my neighborhood or my work place regarding breast cancer prevention program.**	**( )**	**( )**
**12**	**I have donated money or any resources to help breast cancer prevention program in anywhere such as health center, work place or NGOs.**	**( )**	**( )**
**Level 3 (13&14), Level 4 (15,16,17&18) and Level 5(19&20)**			
**13**	**I have taken an active part in organized group activities to carrying out breast cancer prevention programs.**	**( )**	**( )**
**14**	**I have joined organized committees for voluntary work about how breast cancer prevention program should be run.**	**( )**	**( )**
**15**	**I have evaluated and organized the community activities about breast cancer prevention program voluntarily.**	**( )**	**( )**
**16**	**I have encouraged others to join in a breast cancer prevention program group.**	**( )**	**( )**
**17**	**I have asked health staff agencies or government organization to provide the resources or materials which can help breast cancer prevention program.**	**( )**	**( )**
**18**	**I have organized individuals or groups to take greater control over breast cancer prevention program.**	**( )**	**( )**
**19**	**I have participated in planning program to identify the solution about breast cancer prevention.**	**( )**	**( )**
**20**	**I have made decisions about strategies or addressing the problems that women are faced to in getting breast cancer prevention.**	**( )**	**( )**
